# Correlations of Hepatic Hemodynamics, Liver Function, and Fibrosis Markers in Nonalcoholic Fatty Liver Disease: Comparison with Chronic Hepatitis Related to Hepatitis C Virus

**DOI:** 10.3390/ijms17091545

**Published:** 2016-09-14

**Authors:** Ryuta Shigefuku, Hideaki Takahashi, Hiroyasu Nakano, Tsunamasa Watanabe, Kotaro Matsunaga, Nobuyuki Matsumoto, Masaki Kato, Ryo Morita, Yousuke Michikawa, Tomohiro Tamura, Tetsuya Hiraishi, Nobuhiro Hattori, Yohei Noguchi, Kazunari Nakahara, Hiroki Ikeda, Toshiya Ishii, Chiaki Okuse, Shigeru Sase, Fumio Itoh, Michihiro Suzuki

**Affiliations:** 1 Division of Gastroenterology and Hepatology, St. Marianna University School of Medicine, Kanagawa, Kawasaki 216-8511, Japan; hide-bo@marianna-u.ac.jp (H.T.); h-nakano@marianna-u.ac.jp (H.N.); twatanab@marianna-u.ac.jp (T.W.); kotarom@marianna-u.ac.jp (K.M.); nobu1020@marianna-u.ac.jp (N.M.); masaki0801_3@marianna-u.ac.jp (M.K.); r2morita@marianna-u.ac.jp (R.M.); y2michikawa@marianna-u.ac.jp (Y.M.); t2tamura@marianna-u.ac.jp (T.T.); t2hiraishi@marianna-u.ac.jp (T.H.); hattyorina.224@gmail.com (N.H.); y2noguchi@marianna-u.ac.jp (Y.N.); nakahara@marianna-u.ac.jp (K.N.); ikedahi@marianna-u.ac.jp (H.I.); t2ishii@marianna-u.ac.jp (T.I.); c2okuse@marianna-u.ac.jp (C.O.); fitoh@marianna-u.ac.jp (F.I.); michstmu@marianna-u.ac.jp (M.S.); 2 Division of Gastroenterology, St. Marianna University School of Medicine, Yokohama City Seibu Hospital, Kanagawa, Yokohama 241-0811, Japan; 3 Division of Gastroenterology and Hepatology, Department of Internal Medicine, Kawasaki Municipal Tama Hospital, Kanagawa, Kawasaki 214-8525, Japan; 4 Anzai Medical Company, Ltd., Tokyo 141-0033, Japan; sase@anzai-med.co.jp

**Keywords:** nonalcoholic steatohepatitis, chronic hepatitis C, liver function, hepatic hemodynamics, WFA^+^-M2BP

## Abstract

The progression of chronic liver disease differs by etiology. The aim of this study was to elucidate the difference in disease progression between chronic hepatitis C (CHC) and nonalcoholic fatty liver disease (NAFLD) by means of fibrosis markers, liver function, and hepatic tissue blood flow (TBF). Xenon computed tomography (Xe-CT) was performed in 139 patients with NAFLD and 152 patients with CHC (including liver cirrhosis (LC)). The cutoff values for fibrosis markers were compared between NAFLD and CHC, and correlations between hepatic TBF and liver function tests were examined at each fibrosis stage. The cutoff values for detection of the advanced fibrosis stage were lower in NAFLD than in CHC. Although portal venous TBF (PVTBF) correlated with liver function tests, PVTBF in initial LC caused by nonalcoholic steatohepatitis (NASH-LC) was significantly lower than that in hepatitis C virus (C-LC) (*p* = 0.014). Conversely, the liver function tests in NASH-LC were higher than those in C-LC (*p* < 0.05). It is important to recognize the difference between NAFLD and CHC. We concluded that changes in hepatic blood flow occurred during the earliest stage of hepatic fibrosis in patients with NAFLD; therefore, patients with NAFLD need to be followed carefully.

## 1. Introduction

Nonalcoholic fatty liver disease (NAFLD) and nonalcoholic steatohepatitis (NASH) are increasingly recognized as common clinicopathological entities that occur in individuals without significant alcohol use [1]. The former is believed to have a benign clinical course, whereas the latter represents a form of liver injury that carries a risk for progressive fibrosis, liver cirrhosis (LC), and hepatocellular carcinoma (HCC) [2,3]. Due to the obesity epidemic and the increasing prevalence of metabolic syndrome, NAFLD and its progressive form, NASH, are seen more commonly in different parts of the world [4,5]. NAFLD has become a serious public health issue not only in Western countries, but also in many Asian countries, including Japan [6,7,8]. NASH is characterized by parenchymal injuries, including macrovesicular steatosis, ballooning degeneration, Mallory-Denk bodies, and inflammation in hepatic lobes [9]. On the other hand, chronic hepatitis C (CHC) is characterized by portal tract infiltration of dense aggregates of lymphocytes with follicle formation, and mild macrovesicular steatosis can be seen in lobules, particular in periportal hepatocytes [10,11]. Thus, the manner of fibrosis progression in NASH is different from that in CHC. Although there is currently no validated test involving serum biomarkers available to diagnose NASH, and histologic evaluation with a liver biopsy remains the gold standard, and screening for fibrosis is recommended in patients with suspected NASH. However, liver biopsy has some clinical problems related to its invasiveness and complications. On the other hand, there are validated tests with serum biomarkers available to diagnose the stage of hepatic fibrosis (e.g., Wisteria floribunda agglutinin positive Mac-2-binding protein (WFA^+^-M2BP), hyaluronic acid (HA), 7S domain of type IV collagen, tissue inhibitor of metalloproteinase-1 (TIMP-1), type III procollagen N peptide (PIIIP), FIB4-index, etc.). Recently, WFA^+^-M2BP has been reported to be a useful marker for staging in patients with NAFLD [12] and CHC [13,14]. Especially in CHC patients, WFA^+^-M2BP can be a useful surrogate marker not only as a fibrotic marker, but also for the risk of HCC development [13,14]. However, there are few reports about WFA^+^-M2BP on the basis of the etiology of chronic liver disease (CLD).

Since the liver receives blood flow from both the portal vein and hepatic artery, which account for 70% and 30%, respectively, this double blood supply mechanism is a specific characteristic of the liver. The portal vein receives the blood supply from the intestine, which engages in metabolism as a functional vessel. Xenon-CT has been established as a non-invasive technique to visualize tissue blood flow (TBF) in the neurosurgical field [15,16]. Xe-CT can also be applied to obtain separate measurements of hepatic arterial and venous blood flow to detect changes in hepatic blood flow (HBF). We previously reported that PVTBF and total hepatic TBF (THTBF) decrease with the progression of liver fibrosis in patients with CHC [17,18] and NAFLD [19,20]. However, few reports have addressed the association between HBF and liver function; no report has examined the progression of liver function according to the etiology of CLD. Moreover we previously reported that hepatic TBF in patients with liver cirrhosis varied according to the etiology of the disease and there is a close correlation between liver function and hepatic blood flow in patients with alcoholic liver cirrhosis [21,22]. In the present study, we investigated the difference in the fibrosis markers between patients with initial chronic hepatitis and those with advanced chronic hepatitis in NASH and CH-C. Furthermore, we attempted to clarify the relationship between hepatic TBF and liver function. It is extremely important to understand the characteristics of CLD progression for the management and treatment of CLD. The aim of this study was to elucidate the difference in disease progression between CHC and NAFLD in CLD by comparing the cutoff values for fibrosis markers and the associations of liver function and HBF.

## 2. Results

### 2.1. The Cutoff Value and Diagnostic Ability of Each Fibrosis Marker in NAFLD Patients

The receiver operating characteristic (ROC) curve and the area under the ROC (AUC) for each fibrosis marker predict definitive advanced fibrosis. The AUC values of WFA^+^-M2BP, TIMP-1, HA, PIIIP, platelet count (Plt), FIB4-index, aspartate aminotransferase-platelet index (APRI), AST/ALT ratio, and ICG-R_15_ were 0.70, 0.50, 0.87, 0.58, 0.74, 0.77, 0.62, 0.75, and 0.74, respectively (Table 1).

### 2.2. The Cutoff Value and Diagnostic Ability of Each Fibrosis Marker in CHC Patients

The ROC curve and the area under the ROC (AUC) for each fibrosis marker predict definitive advanced fibrosis. The AUC values of WFA^+^-M2BP, TIMP-1, HA, PIIIP, Plt, FIB4-index, APRI, AST/ALT ratio, and ICG-R_15_ were 0.89, 0.84, 0.87, 0.71, 0.82, 0.87, 0.82, 0.62, and 0.86, respectively (Table 2).

### 2.3. Liver Function and Hepatic TBF in Each Stage of NAFLD Patients

Liver function and hepatic TBF in each stage of NAFLD patients are shown in Table 3. With fibrosis progression, Alb, ChE, TC, PT, Plt, PVTBF, and THTBF decreased significantly (*p* < 0.001, *r* = −0.47; *p* < 0.001, *r* = −0.52; *p* < 0.01, *r* = −0.26; *p* < 0.001, *r* = −0.69; *p* < 0.001, *r* = −0.66; *p* < 0.001, *r* = −0.32; *p* < 0.01, *r* = −0.22, respectively). On the other hand, with fibrosis progression, ICG-R_15_, HA, and WFA^+^-M2BP increased significantly (*p* < 0.001, *r* = 0.58; *p* < 0.001, *r* = 0.78; *p* < 0.001, *r* = 0.50, respectively).

### 2.4. Liver Function and Hepatic TBF in Each Stage of CHC Patients

Liver function and hepatic TBF in each stage of CHC patients are shown in Table 4. With fibrosis progression, Alb, Ch-E, TC, PT, Plt, PVTBF, and THTBF decreased significantly (*p* < 0.001, *r* = −0.67; *p* < 0.001, *r* = −0.65; *p* < 0.001, *r* = −0.64; *p* < 0.001, *r* = −0.69; *p* < 0.001, *r* = −0.74; *p* < 0.001, *r* = −0.56; *p* < 0.001, *r* = −0.48, respectively). On the other hand, with fibrosis progression, ICG-R_15_, HA, and WFA^+^-M2BP increased significantly (*p* < 0.001, *r* = 0.39; *p* < 0.001, *r* = 0.76; *p* < 0.001, *r* = 0.62, respectively).

### 2.5. Correlation between Hepatic TBF and Liver Function in NAFLD Patients

Correlations between hepatic TBF, as measured by Xe-CT and liver function in NAFLD patients, are shown in Table 5. There were significant correlations between PVTBF and Alb, ChE, TC, PT, ICG-R_15_, HA, and Plt (*p* < 0.001, *r* = 0.53; *p* < 0.001, *r* = 0.46; *p* < 0.001, *r* = 0.29; *p* < 0.001, *r* = 0.40; *p* < 0.001, *r* = −0.25; *p* < 0.05, *r* = −0.17; *p* < 0.01, *r* = 0.25, respectively). There were also significant correlations between HATBF and ChE and HA (*p* < 0.05, *r* = 0.21; *p* < 0.05, *r* = 0.21, respectively). There were significant correlations between THTBF and ChE, TC, and ICG-R_15_ (*p* < 0.001, *r* = 0.39; *p* < 0.001, *r* = 0.34; *p* < 0.05, *r* = 0.21, respectively). There were significant correlations between the P/A ratio and ChE, TC, and PT (*p* < 0.001, *r* = 0.37; *p* < 0.001, *r* = 0.42; *p* < 0.05, *r* = 0.17, respectively) (Table 5).

### 2.6. Correlation between Hepatic Blood Flow and Liver Function in CHC Patients

Correlations between hepatic blood flow and liver function in CHC patients are shown in Table 6. There were significant correlations between PVTBF and Alb, ChE, TC, PT, ICG-R_15_, HA, and Plt (*p* < 0.001, *r* = 0.50; *p* < 0.001, *r* = 0.66; *p* < 0.001, *r* = 0.66; *p* < 0.001, *r* = 0.70; *p* < 0.001, *r* = −0.36; *p* < 0.001, *r* = 0.37; *p* < 0.001, *r* = 0.37, respectively). There was also a significant correlation between HATBF and PT (*p* < 0.05, *r* = 0.18). There were significant correlations between THTBF and Alb, ChE, TC, PT, and Plt (*p* < 0.001, *r* = 0.42; *p* < 0.001, *r* = 0.55; *p* < 0.001, *r* = 0.67; *p* < 0.001, *r* = 0.37; *p* < 0.001, *r* = 0.35, respectively). There were significant correlations between the P/A ratio and Alb and ChE (*p* < 0.01, *r* = 0.21; *p* < 0.001, *r* = 0.34; *p* < 0.01, *r* = 0.27, respectively) (Table 6).

### 2.7. Comparison of Each TBF at Initial LC (Child-Pugh A) in NASH-LC and C-LC

**Figure 1:**
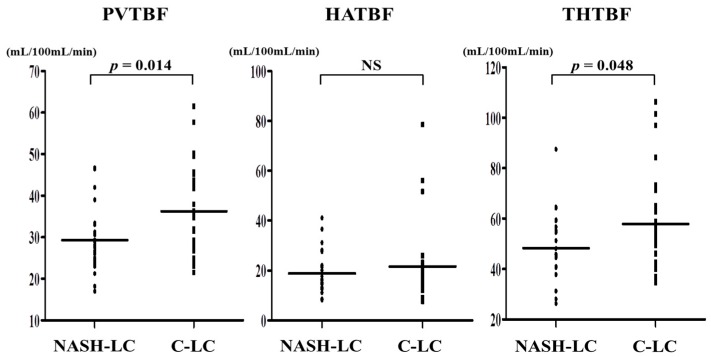
Comparison of each TBF at initial LC (Child-Pugh A) in NASH-LC and C-LC. PVTBF and THTBF are significantly lower in NASH-LC than in C-LC (*p* = 0.014, *p* = 0.048, respectively). HATBF is not significantly different between the LC groups. NS: not significant; PVTBF: portal venous tissue blood flow; HATBF: hepatic arterial tissue blood flow; THTBF: total hepatic tissue blood flow; NASH-LC: liver cirrhosis related to nonalcoholic steatohepatitis; C-LC: liver cirrhosis related to hepatitis C virus.

PVTBF and THTBF were significantly lower in NASH-LC than in C-LC (*p* = 0.014, *p* = 0.048, respectively). Hepatic arterial TBF (HATBF) did not differ significantly between the groups (Figure 1).

### 2.8. Comparison of Each Liver Function Test at Initial LC (Child-Pugh A) in NASH-LC and C-LC

**Figure 2:**
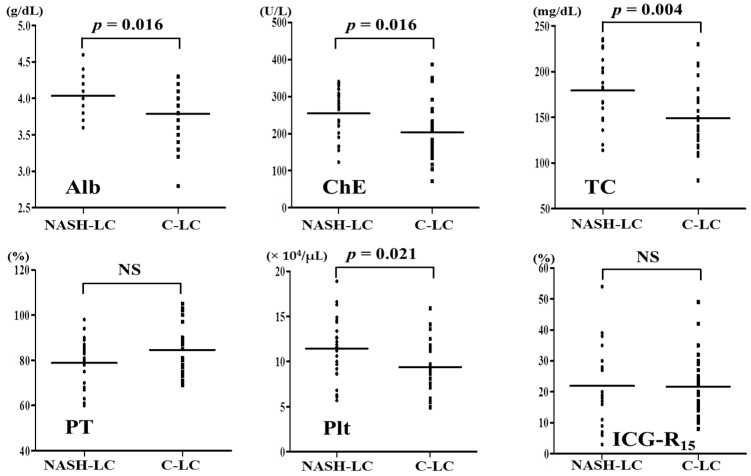
Comparison of each liver function test at initial LC (Child-Pugh A) in NASH-LC and C-LC. Albumin, cholinesterase, total cholesterol, and platelet count are significantly higher in NASH-LC than in C-LC (*p* = 0.016, *p* = 0.016, *p* = 0.004, *p* = 0.021, respectively). NS: not significant; NASH-LC: liver cirrhosis related to nonalcoholic steatohepatitis; C-LC: liver cirrhosis related to hepatitis C virus; Alb: albumin; ChE: cholinesterase; TC: total cholesterol; PT: prothrombin time; Plt: platelet count; ICG-R_15_: retention rate of indocyanine green 15 min after administration.

Alb, Ch-E, TC, and Plt were significantly higher in NASH-LC than in C-LC (*p* = 0.016, *p* = 0.016, *p* = 0.004, *p* = 0.021, respectively). PT and ICG-R_15_ were not significantly different between the groups (Figure 2).

### 2.9. Comparison of Typical Cases at Initial LC (Child-Pugh A) in NASH-LC and C-LC

**Figure 3:**
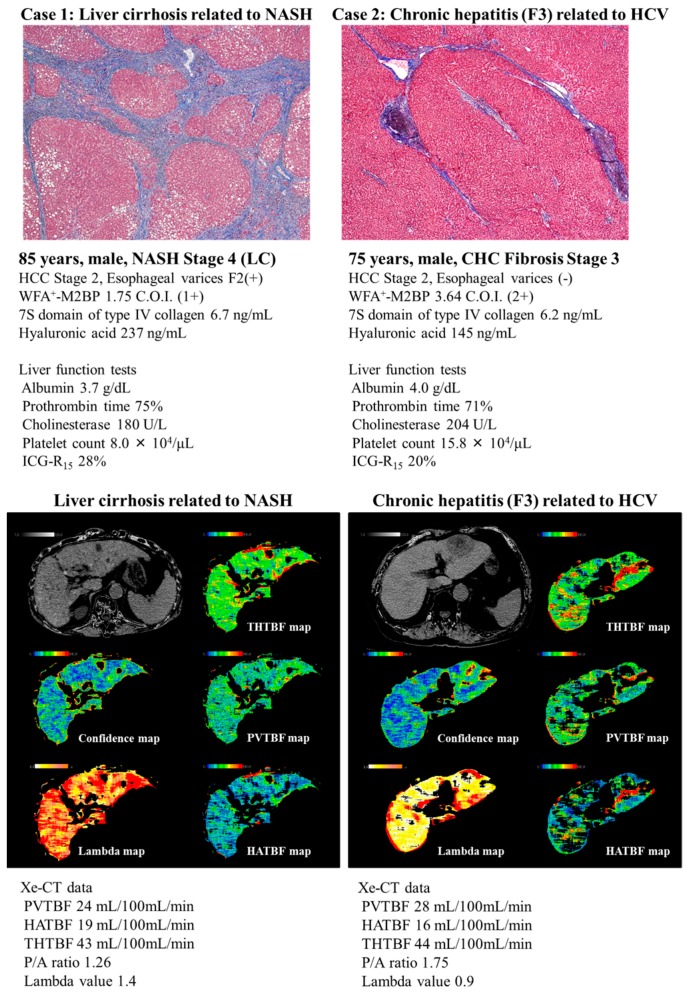
Cases of advanced fibrosis stage in NASH and CHC. Figure 3 shows cases of the advanced fibrosis stage in NASH and CHC. An 85-year-old Japanese man (case 1) was pathologically diagnosed with Stage 4 NASH (Brunt’s classification [23]). His clinical features were also obviously LC-like (e.g., thrombocytopenia, hepatocellular carcinoma, and esophagogastric varices). His fibrosis markers were increased to reflect advanced liver fibrosis. A 75-year-old Japanese man (case 2) was pathologically diagnosed with stage 3 CHC (Desmet’s classification [24]). In case 2, TBF was evaluated in the whole liver excluding the region of hepatocellular carcinoma. The WFA^+^-M2BP of the NASH-LC case was significantly lower than that of the CHC case. TBF was evaluated in both cases. PVTBF and the P/A ratio are lower in NASH-LC (case 1) than in CHC (case 2).

Figure 3 shows cases of the advanced fibrosis stage in NASH and CHC. An 85-year-old Japanese man (case 1) was pathologically diagnosed with Stage 4 NASH (Brunt’s classification [23]). His clinical features were also obviously LC-like (e.g., thrombocytopenia, HCC, and esophagogastric varices). His fibrosis markers were increased, reflecting advanced liver fibrosis. A 75-year-old Japanese man (case 2) was pathologically diagnosed with stage 3 CHC (Desmet’s classification [24]). The WFA^+^-M2BP was significantly lower in NASH-LC than in CHC (Figure 3). In this way, the cutoff values of fibrosis markers, including WFA^+^-M2BP, might differ by the etiology of liver disease. The present results showed that the cutoff values (WFA^+^-M2BP, TIMP-1, HA, and FIB-4 index) to detect the advanced fibrosis stage were lower in NAFLD than in CHC (Table 1 and Table 2). Furthermore, the diagnostic reliability to detect the advanced fibrosis stage was lower in NAFLD than in CHC (Table 1 and Table 2). The reason for this is that the manner of fibrosis progression differs between NASH and CHC. With fibrosis progression, PVTBF gradually decreases in both CHC and NASH. However, PVTBF decreases at an earlier stage in NAFLD than in CHC. This might be attributed to the different manner of fibrosis between NASH and CHC.

## 3. Discussion

A definite diagnosis of NASH requires liver biopsy, though various non-invasive measures are under development [6]. NASH is characterized by parenchymal injury, including macrovesicular steatosis, ballooning degeneration, Mallory-Denk bodies, and inflammation in hepatic lobes [9]. Fibrosis begins in zone 3 or the centrilobular area of the hepatic lobule. Periportal and bridging fibrosis develop with progression of the disease, and once cirrhosis is established, features of steatohepatitis and perisinusoidal fibrosis may be obscured. It is well known that exercise, itself, is an important factor to treat NASH and, therefore, the role of exercise should be emphasized. Exercise, in fact, improves NASH-related fibrosis markers (collagen 1α1 mRNA, *p* < 0.05 and fibrosis score, *p* < 0.01) and the inflammation score; exercise increases the hepatic stellate cell senescence marker CCN1 [25,26].

On the other hand, fibrosis begins in zone 1 or the periportal area of the hepatic lobule in patients with CHC. CHC is characterized by a portal tract that is infiltrated by dense aggregates of lymphocytes with follicle formation, and mild macrovesicular steatosis can be seen in lobules, particularly in periportal hepatocytes [10,11]. Moreover, it has been reported that daily use of recreational drugs, in particular cannabis, has a deleterious effect on the speed of progression of fibrosis and steatosis in patients suffering from chronic hepatitis C [27]. There are other differences between NAFLD and CHC. Previous reports indicated that at the early stages of CLD the numbers of liver monocytes/macrophages were elevated without the evidence of local proliferation, supporting a role for infiltrating monocyte-derived macrophages in disease progression in patients with both CHC and NAFLD. However, CHC and NAFLD differentially affected the circulating monocyte phenotype, suggesting that unique injury-induced signals may contribute to the intrahepatic monocyte recruitment and the systemic activation state. Moreover, it was also shown that monocyte function was similarly impaired in patients with both CHC and NAFLD, particularly in advanced disease [28]. Thus, the manner of fibrosis progression resulting from inflammation could be different between NASH and CHC.

The results of present study showed the relationship between liver function and PVTBF (Table 5 and Table 6). PVTBF was well correlated with hepatic synthesis capacity, which included Alb, ChE, TC, and PT. The reason why liver function tests in NASH was better than that in CHC is suggested the excess energy intake and lipid hypermetabolism [29]. ICG-R_15_ is the indicator which reflects liver function [30] and the presence of portal hypertension. Furthermore ICG-R_15_ is well correlated to the hepatic tissue blood flow [31]. Lisotti et al. reported that the ICG-R_15_ test is an effective tool for assessment of portal hypertension in patients with compensated cirrhosis [30]. We confirmed that the hemodynamic changes occurred earlier in NAFLD relative to CHC. For example, 15% of ICG-R_15_ correspond to the stage 3 in NASH and LC in CHC (Table 3 and Table 4). Yamazaki reported that the average of ICG-R_15_ was 15.4% in which the presence of esophageal varices cases [32], and their data, supported our results.

Alteration in hepatic microcirculation in human donor livers with steatosis was first reported during organ retrieval before mobilization by Seifalian et al. [33] using laser Doppler flowmetry. A significant decrease in hepatic microcirculation in liver donors with steatosis was observed in comparison with that in normal liver donors [34]. Experimental studies in animal models with fatty liver showed that steatosis led to reduce hepatic blood flow and microcirculation, and that there was an inverse correlation between the degree of steatosis and both total hepatic blood flow and flow in the microcirculation [30]. The severity of fatty infiltration has a greater effect on the microcirculation than on total hepatic blood flow [35,36]. In spite of steatosis alone, hepatic blood flow reduced. Moreover, hepatic blood flow reduced with fibrosis development, in addition to steatosis [37]. Fat-laden hepatocytes are swollen, and in steatohepatitis, further swelling occurs due to the ballooning of hepatocytes, causing sinusoidal distortion, as visualized by in vivo microscopy, reducing intrasinusoidal volume and microcirculation [38].

In addition to steatosis, a mechanism of decreasing portal blood flow other than steatosis has been reported in NAFLD. In livers with perfusion from cafeteria diet-fed rats, there was increased portal pressure and decreased endothelium-dependent vasodilation. This was associated with decreased Akt-dependent endothelial nitric-oxide synthase (eNOS) phosphorylation and NOS activity. They demonstrated in a rat model of the metabolic syndrome that hepatic endothelial dysfunction occurs before the development of fibrosis and inflammation [39]. Ying-Ying Yang et al. reported that hyperleptinemia increases hepatic endocannabinoid production, promotes hepatic fibrogenesis, enhances the hepatic vasoconstrictive response to endothelin-1, and aggravates hepatic microcirculatory dysfunction. These events subsequently increase intrahepatic resistance and portal hypertension in NASH cirrhotic rats [40].

The present data show that the liver function was better in initial NASH-LC than in C-LC. However, because PVTBF was lower in NASH than in C-LC, portal hypertension might occur at an earlier stage in NASH than in CHC. In fact, portal hypertension occurs without LC in NASH [41,42,43]. Mendes et al. investigated the prevalence of portal hypertension in NAFLD patients, and found that clinical signs of portal hypertension, including esophageal varices, splenomegaly, portosystemic encephalopathy, and ascites, were present in 25% of patients at the time of diagnosis. Furthermore, portal hypertension can occur in a small proportion of patients with mild or no fibrosis and is associated with the extent of steatosis [44]. Brunt et al. reported that hepatic fibrosis in NAFLD patients was found in the pericellular space around the central vein and in the presinusoidal region in zone 3 in the early stage [23]. The pericellular fibrosis in the early stage of NAFLD patients may lead to an elevated portal vascular resistance and result in a change of hepatic blood flow [45]. Therefore, we considered that the hemodynamic changes occurred earlier in NAFLD relative to CHC.

In this study, there are two limitations, such as sampling error during liver biopsy and by permeation of Xe gas. Xe-CT cannot monitor the exact result in the patients with chronic lung disease (e.g., chronic obstructive pulmonary disease, lung cancer) and heart failure, because Xe gas is taken up by the lung via the respiratory tract. On the contrary, we believe that there are also many strong points of Xe-CT which objectively and repeatedly measure hepatic blood flow with reproducibility. Moreover, we have safely performed a Xe-CT for patients with acute or chronic renal failure because there are no complications associated with the contrast agent, such as allergic reactions and radiocontrast nephropathy.

Thus, in the present study, the difference between NAFLD and CHC was investigated based on TBF, fibrosis markers, and liver function. In conclusion, compared to C-LC, PVTBF decreased significantly in the Child-Pugh A stage of NASH-LC, indicating that portal hemodynamic changes could occur earlier in NASH-LC without impaired liver function. Therefore, patients with NASH should be monitored carefully for portal hypertensive complications in the early fibrosis stage.

## 4. Materials and Methods

### 4.1. Patients

Between October 2001 and March 2016, 730 patients underwent Xe-CT at the St. Marianna University School of Medicine Hospital. Of the 730 patients, 291 with NAFLD and CHC were enrolled in this study. Liver biopsy was performed for 118 of the 139 NAFLD patients and 106 of the 152 CHC patients. During hospitalization for three days, Xe-CT was performed before or after each liver biopsy. The NAFLD patients included 80 men and 59 women, with a mean age of 53.2 ± 11.2 years and a mean body mass index (BMI) of 28.5 ± 4.9 kg/m^2^. The CHC patients included 75 men and 77 women, with a mean age of 59.9 ± 11.2 years and a mean BMI of 23.2 ± 3.7 kg/m^2^ (Table 7).

The diagnosis of NAFLD was based on: (1) substantial alcohol consumption (>20 g/day for women or >30 g/day for men); (2) pathological findings showing characteristics of NAFLD (large-droplet fat deposits, hepatocyte ballooning, inflammatory cell infiltration, and fibrosis around the central vein); and (3) the exclusion of other liver diseases, such as viral hepatitis, autoimmune liver disease, and drug-induced liver injury. The diagnosis of CHC was based on anti-HCV antibodies and HCV-RNA. Patients were excluded for the presence of other causes of liver disease, acute illness, acute or chronic inflammatory or infective diseases, an end-stage malignant disease, or other confounding conditions. Liver biopsy was performed through the right intercostal space under ultrasonography-guided liver biopsy using a 16-gauge needle biopsy kit (Quick-Core^®^ biopsy needle set; Cook Medical, Bloomington, IN, USA). The aims of liver biopsy were to assess fibrosis and steatosis and to exclude other liver disease. Histological diagnosis was confirmed by two experienced pathologists who were blinded to the clinical data. There were 15 patients with nonalcoholic fatty liver (NAFL) who had no fibrosis and inflammatory cell infiltration. The patients with NASH were evaluated on the basis of Brunt’s classification [21,46,47], while those with CHC were evaluated on the basis of Desmet’s classification [22]. Staging fibrosis in NASH based on Brunt’s classification: Stage 1: zone 3 perivenular perisinusoidal/pericellular fibrosis, focal or extensive; Stage 2: as above with focal or extensive periportal fibrosis; Stage 3: bridging fibrosis, focal or extensive; and Stage 4: cirrhosis. Staging fibrosis in CHC based on Desmet’s classification: Stage 0: lack of fibrosis; Stage 1: fibrosis confined to portal tract; Stage 2: bridging fibrosis; Stage 3: bridging fibrosis with structural distortion; and Stage 4: cirrhosis. Clinical liver cirrhosis was defined by the presence of a portosystemic shunt or ascites.

### 4.2. Xe-CT Theory and Imaging Protocol

As described in previous publications, 25% stable Xe gas was used in conjunction with an AZ-726 Xe gas inhalation system (Anzai Medical, Tokyo, Japan) [48,49]. The wash-in and wash-out periods were both 4 min. The entire liver was CT-scanned at 1-min intervals at four levels, including the porta hepatis (nine scans in total, including the baseline scan). Using an AZ-7000W image processing system (Anzai Medical), PVTBF and HATBF were calculated, and PVTBF and HATBF maps were created. THTBF was calculated as the sum of PVTBF and HATBF, and THTBF maps were also created. The time course change rate for the arterial Xe concentration, which was needed to calculate PVTBF and HATBF, was derived using the time course of the Xe concentration in splenic tissue. An Aquilion CT scanner (Toshiba Medical Systems, Tokyo, Japan) was used, with exposure factors of 120 kV, 150 mA, and 13.8 mGy. All examinations were performed with the patients in the fasting state. Informed consent was obtained from each patient. All study protocols were reviewed and approved by the ethics committee at our institution (approval No. 480, 18 September 2001), and conformed to the ethics guidelines of the 1975 Declaration of Helsinki (Allen, 1991).

### 4.3. Liver Function Tests and Fibrosis Markers

Liver function tests were measured on admission. Liver function tests included the following parameters: albumin (Alb) (g/dL), cholinesterase (ChE) (IU/L), total cholesterol (TC) (mg/dL), prothrombin time (PT) (%), Plt (×10^4^ μL^−1^), hyaluronic acid (HA) (ng/mL), Wisteria floribunda agglutinin positive Mac-2-binding protein (WFA^+^-M2BP) (C.O.I.), and the retention rate of indocyanine green 15 min after administration (ICG-R_15_) (%). ICG (Diagnogreen^®^, Daiichisankyo Pharmaceutical Co., Tokyo, Japan; 0.5 mg/kg body weight) was administered via a peripheral vein, and venous blood was sampled before and 15 min after injection. Specimens were analyzed for ICG concentrations on a spectrophotometer (HITACHI, Tokyo, Japan) at 805 nm.

### 4.4. Measurements of TIMP-1, HA, PIIIP, and WFA^+^-M2BP

For all patients in the cohort, the blood sample was taken on the day of the liver biopsy at the St. Marianna University School of Medicine Hospital. All samples were processed to separate serum and stored at −80 °C. At the time of blood withdrawal, all patients underwent liver biopsy. In this study, 58 samples of NAFLD and 72 samples of CHC were enrolled (Table 7). TIMP-1, HA, and PIIIP were measured using a fully automatic immunoanalyzer (Sysmex Co., Hyogo, Japan). WFA^+^-M2BP quantification was measured based on a lectin-Ab sandwich immunoassay using a fully automatic immunoanalyzer, HISCL-2000i (Sysmex Co., Hyogo, Japan) [50].

### 4.5. Statistical Analysis

Each parameter is expressed as the mean ± standard deviation (SD). Spearman’s rank correlation coefficient was used to examine correlations of TBF with progression of fibrosis. The Pearson product-moment correlation coefficient was used to examine correlations between TBF parameters and liver function tests. To assess the utility of each fibrosis marker to distinguish the advanced fibrosis stage, the sensitivity and specificity were calculated for each value, and then ROC curves were constructed by plotting the sensitivity against the reverse specificity (1-the specificity) for each value. We used Student’s *t*-test, which was two-tailed and performed by the statistical software GraphPad Prism (GraphPad Software, San Diego, CA, USA). *p*-Values of <0.05 were considered significant.

## 5. Conclusions

It is important to recognize the difference between NAFLD and CHC. We concluded that changes in hepatic blood flow occurred during the earliest stage of hepatic fibrosis in patients with NAFLD and, therefore, patients with NAFLD need to be followed carefully.

## Figures and Tables

**Table 1:** The cutoff value and diagnostic ability of each fibrosis marker in NAFLD patients.

NAFLD (*n* = 58)					
Fibrosis Markers		Cutoff	AUROC	Sensitivity	Specificity
WFA^+^-M2BP	C.O.I	1.06	0.70	75	67
TIMP-1	ng/mL	242.0	0.50	50	68
HA	ng/mL	58.9	0.87	80	86
PIIIP	ng/mL	11.4	0.58	50	74
Platelet count	×10^4^/μL	17.7	0.74	67	80
FIB-4 Index	–	1.95	0.77	67	78
APRI	–	3.25	0.62	50	70
AST/ALT ratio	–	0.82	0.75	75	78
ICG-R_15_	%	10.5	0.74	67	64

Stage 0–2 (*n* = 46) vs. Stage 3–4 (*n* = 12). AUROC, area under the receiver operating characteristic curve; NAFLD: nonalcoholic fatty liver disease.

**Table 2:** The cutoff value and diagnostic ability of each fibrosis marker in CHC patients.

CHC (*n* = 72)					
Fibrosis Markers		Cutoff	AUROC	Sensitivity	Specificity
WFA^+^-M2BP	C.O.I	3.28	0.89	84	85
TIMP-1	ng/mL	297.6	0.84	88	72
HA	ng/mL	116.5	0.87	79	79
PIIIP	ng/mL	10.6	0.71	74	64
Platelet count	×10^4^/μL	13.9	0.82	74	75
FIB-4 Index	–	3.19	0.87	89	79
APRI	–	5.41	0.82	79	79
AST/ALT ratio	–	0.76	0.62	63	53
ICG-R_15_	%	11.5	0.86	84	76

Stage 0–2 (*n* = 53) vs. Stage 3–4 (*n* = 19). AUROC, area under the receiver operating characteristic curve; CHC: chronic hepatitis related to hepatitis C virus.

**Table 3:** Liver function and hepatic tissue blood flow in each fibrosis stage in NAFLD.

Fibrosis Stage	NAFL	Stage 1	Stage 2	Stage 3	Initial LC	Advanced LC	*p*-Value *	*r*
	*n* = 15	*n* = 47	*n* = 30	*n* = 15	*n* = 25	*n* = 7		
Alb (g/dL)	4.5 ± 0.3	4.5 ± 0.3	4.4 ± 0.3	4.4 ± 0.3	4.0 ± 0.3	3.1 ± 0.4	<0.001	−0.47
ChE (IU/L)	390.1 ± 107.2	372.8 ± 104.3	346.3 ± 96.0	260.5 ± 149.3	254.8 ± 63.2	155.4 ± 95.7	<0.001	−0.52
TC (mg/dL)	200.8 ± 40.9	200.4 ± 33.5	180.3 ± 62.0	170.3 ± 73.9	179.5 ± 36.3	119.7 ± 39.0	<0.01	−0.26
PT (%)	100.8 ± 5.4	98.9 ± 9.0	96.4 ± 6.5	87.0 ± 10.1	76.4 ± 16.2	57.0 ± 7.7	<0.001	−0.69
ICG-R_15_ (%)	5.9 ± 2.9	9.8 ± 13.0	11.6 ± 6.6	17.7 ± 18.1	21.9 ± 12.5	26.0 ± 8.4	<0.001	0.58
HA (ng/mL)	20.4 ± 16.5	53.4 ± 98.0	63.4 ± 51.3	151.4 ± 98.7	268.9 ± 193.9	347.3 ± 181.9	<0.001	0.78
WFA^+^-M2BP (C.O.I)	0.7 ± 0.3	0.8 ± 0.4	1.2 ± 0.7	1.3 ± 0.6	1.8 ± 1.5	4.4 ± 0.2	<0.001	0.50
Plt (×10^4^/μL)	24.2 ± 4.5	23.7 ± 6.5	20.1 ± 5.2	18.3 ± 7.3	11.5 ± 3.5	5.3 ± 3.0	<0.001	−0.66
PVTBF (mL/100 mL/min)	41.0 ± 6.3	34.3 ± 7.2	33.8 ± 7.0	29.9 ± 6.7	29.3 ± 7.9	29.9 ± 2.4	<0.001	−0.32
HATBF (mL/100 mL/min)	23.8 ± 9.0	21.30 ± 10.2	19.6 ± 10.1	20.5 ± 8.3	18.8 ± 8.6	24.2 ± 6.2	NS	−0.09
THTBF (mL/100 mL/min)	64.9 ± 13.4	55.6 ± 14.0	53.4 ± 14.2	48.1 ± 10.1	46.5 ± 10.9	54.9 ± 9.4	<0.01	−0.22
P/A ratio Fibro	1.9 ± 0.7	1.9 ± 0.7	2.0 ± 0.6	1.6 ± 0.5	1.6 ± 0.5	1.4 ± 0.4	NS	−0.15

* Spearman’s rank correlation coefficient was used to examine correlations of TBF with the progression of fibrosis; NS: not significant; P/A ratio: portal flow/hepatic arterial flow ratio.

**Table 4:** Liver function and hepatic tissue blood flow in each fibrosis stage in CHC.

Fibrosis Stage	Stage 1	Stage 2	Stage 3	Initial LC	Advanced LC	*p*-Value *	*r*
	*n* = 45	*n* = 29	*n* = 21	*n* = 30	*n* = 27		
Alb (g/dL)	4.3 ± 0.3	4.3 ± 0.3	4.1 ± 0.3	3.8 ± 0.8	3.1 ± 0.3	<0.001	−0.67
ChE (IU/L)	329.0 ± 81.4	268.2 ± 74.7	226.0 ± 54.8	202.9 ± 75.4	113.0 ± 40.7	<0.001	−0.65
TC (mg/dL)	182.8 ± 31.0	167.4 ± 34.7	150.7 ± 28.4	149.3 ± 33.9	120.8 ± 36.0	<0.001	−0.64
PT (%)	96.2 ± 8.7	88.0 ± 8.8	81.5 ± 10.9	86.4 ± 10.9	64.0 ± 9.7	<0.001	−0.69
ICG-R15 (%)	7.4 ± 3.7	13.5 ± 9.6	14.1 ± 5.1	21.7 ± 10.0	34.2 ± 11.2	<0.001	0.39
HA (ng/mL)	54.8 ± 60.3	156.1 ± 203.4	275.8 ± 202.2	425.1 ± 362.7	565.2 ± 370.7	<0.001	0.76
WFA^+^-M2BP (C.O.I)	1.5 ± 1.5	1.9 ± 1.9	4.5 ± 4.5	4.0 ± 4.2	5.6 ± 5.8	<0.001	0.62
Plt (×10^4^/μL)	18.4 ± 4.8	15.1 ± 5.0	12.1 ± 3.6	9.3 ± 3.6	7.3 ± 2.7	<0.001	−0.74
PVTBF (mL/100 mL/min)	48.8 ± 13.9	40.4 ± 13.5	36.3 ± 7.9	36.3 ± 11.2	26.6 ± 7.5	<0.001	−0.56
HATBF (mL/100 mL/min)	26.1 ± 14.2	21.9 ± 8.6	20.5 ± 11.7	21.6 ± 15.0	21.5 ± 13.1	NS	−0.18
THTBF (mL/100 mL/min)	74.9 ± 21.8	62.4 ± 16.9	56.8 ± 12.7	54.6 ± 15.1	48.3 ± 14.2	<0.001	−0.48
P/A ratio	2.1 ± 1.0	1.9 ± 0.9	1.9 ± 1.0	1.9 ± 0.8	1.6 ± 0.9	NS	−0.17

* Spearman’s rank correlation coefficient was used to examine correlations of TBF with the progression of fibrosis; NS: not significant; P/A ratio: portal flow/hepatic arterial flow ratio.

**Table 5:** Correlations of liver function and hepatic tissue blood flow in NAFLD.

TBF	PVTBF		HATBF		THTBF		P/A Ratio	
	*p*-Value *	*r*	*p*-Value *	*r*	*p*-Value *	*r*	*p*-Value *	*r*
Alb (g/dL)	<0.001	0.53	NS	−0.04	NS	0.02	NS	0.14
ChE (IU/L)	<0.001	0.46	<0.05	0.21	<0.001	0.39	<0.001	0.37
TC (mg/dL)	<0.001	0.29	NS	0.16	<0.001	0.34	<0.001	0.42
PT (%)	<0.001	0.40	NS	−0.06	NS	0.02	<0.05	0.17
ICG-R_15_ (%)	<0.01	−0.25	NS	0.09	<0.05	0.21	NS	−0.01
HA (ng/mL)	<0.05	−0.17	<0.05	0.21	NS	0.03	NS	0.06
Plt (×10^4^/μL)	<0.01	0.25	NS	−0.02	NS	0.07	NS	0.05

* The Pearson product-moment correlation coefficient was used to examine correlations between TBF parameters and liver function tests. TBF: tissue blood flow; NS: not significant; P/A ratio: portal flow/hepatic arterial flow ratio.

**Table 6:** Correlations of liver function and hepatic tissue blood flow in CHC.

TBF	PVTBF		HATBF		THTBF		P/A Ratio	
	*p*-Value *	*r*	*p*-Value *	*r*	*p*-Value *	*r*	*p*-Value *	*r*
Alb (g/dL)	<0.001	0.50	NS	−0.05	<0.001	0.42	<0.01	0.21
ChE (IU/L)	<0.001	0.66	NS	0.12	<0.001	0.55	<0.001	0.34
TC (mg/dL)	<0.001	0.66	NS	0.10	<0.001	0.67	<0.01	0.27
PT (%)	<0.001	0.70	<0.05	0.18	<0.001	0.37	NS	0.09
ICG-R_15_ (%)	<0.001	−0.36	NS	0.07	NS	0.10	NS	0.05
HA (ng/mL)	<0.001	0.37	NS	−0.13	NS	−0.10	NS	0.06
Plt (×10^4^/μL)	<0.001	0.37	NS	0.07	<0.001	0.35	NS	0.07

* The Pearson product-moment correlation coefficient was used to examine correlations between TBF parameters and liver function tests; TBF: tissue blood flow; NS: not significant; P/A ratio: portal flow/hepatic arterial flow ratio.

**Table 7:** Characteristics of patients.

Group	NAFLD	CHC
Number of cases	139	152
Sex (Male/Female)	80/59	75/77
Age (years)	53.2 ± 11.2	59.9 ± 11.2 *
BMI (kg/m^2^)	28.5 ± 4.9	23.2 ± 3.7 *
Staging for fibrosis	NAFL/NASH Stage 1/2/3/4 + Child A/Child B,C	Stage 0,1/2/3/4 + Child A/Child B,C
	(Brunt’s classification) 15/47/30/15/25/7	(Desmet’s classification) 45/29/21/30/27
Number of cases **	58	72
Mild fibrosis group (Stage 0–2)	46	53
Advanced fibrosis group (Stage 3–4)	12	19

* *p* < 0.05 (unpaired *t*-test); ** In this study, 58 samples of NAFLD and 72 samples of CH-C were enrolled. The blood sample was taken on the day of the liver biopsy.

## Abbreviations

Alb	albumin
APRI	aspartate aminotransferase-platelet index
CHC	chronic hepatitis related to hepatitis C virus
ChE	cholinesterase
CLD	chronic liver disease
CT	computed tomography
HA	hyaluronic acid
HATBF	hepatic arterial tissue blood flow
HBF	hepatic blood flow
HCV	hepatitis C virus
ICG-R_15_	retention rate of indocyanine green 15 min after administration
LC	liver cirrhosis
MRI	magnetic resonance imaging
NAFLD	nonalcoholic fatty liver disease
NASH	nonalcoholic steatohepatitis
Plt	platelet count
PT	prothrombin time
P/A	portal flow / hepatic arterial flow ratio
PVTBF	portal venous tissue blood flow
PIIIP	type III procollagen N peptide
ROI	region of interest
TC	total cholesterol
TBF	tissue blood flow
THTBF	total hepatic tissue blood flow
TIMP-1	tissue inhibitor of metalloproteinase-1
US	ultrasonography
WFA^+^-M2BP	Wisteria floribunda agglutinin positive Mac-2-binding protein
Xe-CT	Xenon computed tomography

## References

[1] Ludwig J., Viggiano T.R., McGill D.B., Oh B.J. (1980). Nonalcoholic steatohepatitis: Mayo clinic experiences with a hitherto unnamed disease. Mayo Clin. Proc..

[2] Matteoni C.A., Younossi Z.M., Gramlich T., Boparai N., Liu Y.C., Mc Cullough A.J. (1999). Nonalcoholic fatty liver disease: A spectrum of clinical and pathological severity. Gastroenterology.

[3] Farrell G.C., Larter C.Z. (2006). Nonalcoholic fatty liver disease: From steatosis to cirrhosis. Hepatology.

[4] Sayiner M., Koenig A., Henry L., Younossi Z.M. (2016). Epidemiology of nonalcoholic fatty liver disease and nonalcoholic steatohepatitis in the United States and the rest of the world. Clin. Liver Dis..

[5] Sherif Z.A., Saeed A., Ghavimi A., Nouraie S.-M., Laiyemo A.O., Brim H., Ashktorab H. (2016). Global epidemiology of nonalcoholic fatty liver disease and perspectives on US minority populations. Dig. Dis. Sci..

[6] Watanabe S., Hashimoto E., Ikejima K., Uto H., Ono M., Sumida Y., Seike M., Takei Y., Takehara T., Tokushige K. (2015). Evidence-based clinical practice guidelines for nonalcoholic fatty liver disease/nonalcoholic steatohepatitis. J. Gastroenterol..

[7] Eguchi Y., Hyogo H., Ono M., Mizuta T., Ono N., Fujimoto K., Chayama K., Saibara T. (2012). JSG-NAFLD. Prevalence and associated metabolic factors of nonalcoholic fatty liver disease in the general population from 2009 to 2010 in Japan: A multicenter large retrospective study. J. Gastroenterol..

[8] Farrell G.C., Wong V.W., Chitturi S. (2013). NAFLD in Asia—As common and important as in the West. Nat. Rev. Gastroenterol. Hepatol..

[9] Brunt E.M. (2004). Nonalcoholic steatohepatitis. Semin. Liver Dis..

[10] Freni M.A., Artuso D., Gerken G., Spanti C., Marafioti T., Alessi N., Spadaro A., Ajello A., Ferraù O. (1995). Focal lymphocytic aggregates in chronic hepatitis C: Occurrence, immunohistochemical characterization, and relation to markers of autoimmunity. Hepatology.

[11] Wong V.S., Wight D.G., Palmer C.R., Alexander G.J. (1996). Fibrosis and other histological features in chronic hepatitis C virus infection: A statistical model. J. Clin. Pathol..

[12] Abe M., Miyake T., Kuno A., Imai Y., Sawai Y., Hino K., Hara Y., Hige S., Sakamoto M., Yamada G. (2015). Association between *Wisteria floribunda* agglutinin-positive Mac-2 binding protein and the fibrosis stage of non-alcoholic fatty liver disease. J. Gastroenterol..

[13] Yamasaki K., Tateyama M., Abiru S., Komori A., Nagaoka S., Saeki A., Hashimoto S., Sasaki R., Bekki S., Kugiyama Y. (2014). Elevated serum levels of *Wisteria floribunda* agglutinin-positive human Mac-2 binding protein predict the development of hepatocellular carcinoma in hepatitis C patients. Hepatology.

[14] Tamaki N., Kurosaki M., Kuno A., Korenaga M., Togayachi A., Gotoh M., Nakakuki N., Takada H., Matsuda S., Hattori N. (2015). *Wisteria floribunda* agglutinin positive human Mac-2-binding protein as a predictor of hepatocellular carcinoma development in chronic hepatitis C patients. Hepatol. Res..

[15] Johnson D.W., Stringer W.A., Marks M.P., Yonas H., Good W.F., Gur D. (1991). Stable xenon CT cerebral blood flow imaging: Rationale for and role in clinical decision making. Am. J. Neuroradiol..

[16] Gur D., Good W.F., Wolfson S.K., Yonas H., Shabason L. (1982). In vivo mapping of local cerebral blood flow by xenonenhanced computed tomography. Science.

[17] Ikeda H., Suzuki M., Kobayashi M., Takahashi H., Matsumoto N., Maeyama S., Iino S., Sase S., Itoh F. (2007). Xenon computed tomography shows hemodynamic change during the progression of chronic hepatitis C. Hepatol. Res..

[18] Shigefuku R., Takahashi H., Kato M., Yoshida Y., Suetani K., Noguchi Y., Hatsugai M., Nakahara K., Ikeda H., Kobayashi M. (2014). Evaluation of hepatic tissue blood flow using xenon computed tomography with fibrosis progression in nonalcoholic fatty liver disease: Comparison with chronic hepatitis C. Int. J. Mol. Sci..

[19] Shigefuku R., Takahashi H., Kobayashi M., Ikeda H., Matsunaga K., Okuse C., Matsumoto N., Maeyama S., Sase S., Suzuki M. (2012). Pathophysiological analysis of nonalcoholic fatty liver disease by evaluation of fatty liver changes and blood flow using xenon computed tomography: Can early-stage nonalcoholic steatohepatitis be distinguished from simple steatosis?. J. Gastroenterol..

[20] Kobayashi M., Suzuki M., Ikeda H., Takahashi H., Matsumoto N., Maeyama S., Sase S., Iino S., Itoh F. (2009). Assessment of hepatic steatosis and hepatic tissue blood flow by xenon computed tomography in nonalcoholic steatohepatitis. Hepatol. Res..

[21] Takahashi H., Suzuki M., Ikeda H., Kobayashi M., Sase S., Yotsuyanagi H., Maeyama S., Iino S., Itoh F. (2010). Evaluation of quantitative portal venous, hepatic arterial, and total hepatic tissue blood flow using xenon CT in alcoholic liver cirrhosis—Comparison with liver cirrhosis related to hepatitis C virus and nonalcoholic steatohepatitis. Alcohol. Clin. Exp. Res..

[22] Takahashi H., Shigefuku R., Yoshida Y., Ikeda H., Matsunaga K., Matsumoto N., Okuse C., Sase S., Itoh F., Suzuki M. (2014). Correlation between hepatic blood flow and liver function in alcoholic liver cirrhosis. World J. Gastroenterol..

[23] Brunt E.M., Janney C.G., di Bisceglie A.M., Neuschwander-Tetri B.A., Bacon B.R. (1999). Non-alcoholic steatohepatitis: A proposal for grading and staging the histological lesions. Am. J. Gastroenterol..

[24] Desmet V.J., Gerber M., Hoofnagle J.H., Manns M., Scheuer P.J. (1994). Classification of chronic hepatitis: Diagnosis, grading and staging. Hepatology.

[25] Linden M.A., Sheldon R.D., Meers G.M., Ortinau L.C., Morris E.M., Booth F.W., Kanaley J.A., Vieira-Potter V.J., Sowers J.R., Ibdah J.A. (2016). Aerobic exercise training in the treatment of NAFLD related fibrosis. J. Physiol..

[26] Finelli C., Tarantino G. (2012). Have guidelines addressing physical activity been established in nonalcoholic fatty liver disease?. World J. Gastroenterol..

[27] Tarantino G., Citro V., Finelli C. (2014). Recreational drugs: A new health hazard for patients with concomitant chronic liver diseases. J. Gastrointest. Liver Dis..

[28] Gadd V.L., Patel P.J., Jose S., Horsfall L., Powell E.E., Irvine K.M. (2016). Altered peripheral blood monocyte phenotype and function in chronic liver disease: Implications for hepatic recruitment and systemic inflammation. PLoS ONE.

[29] Neuschwander-Tetri B.A. (2010). Hepatic lipotoxicity and the pathogenesis of nonalcoholic steatohepatitis: The central role of nontriglyceride fatty acid metabolites. Hepatology.

[30] Makuuchi M., Kosuge T., Takayama T., Yamazaki S., Kakazu T., Miyagawa S., Kawasaki S. (1993). Surgery for small liver cancers. Semin. Surg. Oncol..

[31] Lisotti A., Azzaroli F., Buonfiglioli F., Montagnani M., Cecinato P., Turco L., Calvanese C., Simoni P., Guardigli M., Arena R. (2014). Indocyanine Green retention test as a noninvasive marker of portal hypertension and esophageal varices in compensated liver cirrhosis. Hepatology.

[32] Yamazaki S., Takayama N., Nakamura M., Higaki T., Matsuoka S., Mizuno S., Moriyama M. (2014). Prophylactic impact of endoscopic treatment for esophageal varices in liver resection: A prospective study. J. Gastroenterol..

[33] Seifalian A.M., Mallet S.V., Rolles K., Davidson B.R. (1997). Hepatic microcirculation during human orthotopic liver transplantation. Br. J. Surg..

[34] Seifalian A.M., Chidambaram V., Rolles K., Davidson B.R. (1998). In vivo demonstration of impaired microcirculation in steatotic human liver grafts. Liver Transplant. Surg..

[35] Seifalian A.M., Piasecki C., Agarwal A., Davidson B.R. (1999). The effect of graded steatosis on flow in the hepatic parenchymal microcirculation. Transplantation.

[36] Samia I., Wenxuan Y., Winslet M.C., Alexander M., Seifalian A.M. (2003). Impairment of hepatic microcirculation in fatty liver. Microcirculation.

[37] Hayashi N., Kasahara A., Kurosawa K., Sasaki Y., Fusamoto H., Sato N., Kamada T. (1985). Oxygen supply to the liver in patients with alcoholic liver disease assessed by organ-reflectance spectrophotometry. Gastroenterology.

[38] Farrell G.C., Teoh N.C., McCuskey R.S. (2008). Hepatic microcirculation in fatty liver disease. Anat. Rec. (Hoboken).

[39] Pasarín M., La Mura V., Gracia-Sancho J., García-Calderó H., Rodríguez-Vilarrupla A., García-Pagán J.C., Bosch J., Abraldes J.G. (2012). Sinusoidal endothelial dysfunction precedes inflammation and fibrosis in a model of NAFLD. PLoS ONE.

[40] Yang Y.-Y., Tsai T.-H., Huang Y.-T., Lee T.-Y., Chan C.-C., Lee K.-C., Lin H.-C. (2012). Hepatic endothelin-1 and endocannabinoids-dependent effects of hyperleptinemia in nonalcoholic steatohepatitis-cirrhotic Rats. Hepatology.

[41] Francque S., Verrijken A., Mertens I., Hubens G., van Marck E., Pelckmans P., van Gaal L., Michielsen P. (2010). Noncirrhotic human nonalcoholic fatty liver disease induces portal hypertension in relation to the histological degree of steatosis. Eur. J. Gastroenterol. Hepatol..

[42] Hashimoto E., Yatsuji S., Kaneda H., Yoshioka Y., Taniai M., Tokushige K., Shiratori K. (2005). The characteristics and natural history of Japanese patients with nonalcoholic fatty liver disease. Hepatol. Res..

[43] Nakamura S., Konishi H., Kishino M., Yatsuji S., Tokushige K., Hashimoto E., Shiratori K. (2008). Prevalence of esophagogastric varices in patients with nonalcoholic steatohepatitis. Hepatol. Res..

[44] Mendes F.D., Suzuki A., Sanderson S.O., Lindor K.D., Angulo P. (2012). Prevalence and indicators of portal hypertension in patients with nonalcoholic fatty liver disease. Clin. Gastroenterol. Hepatol..

[45] Hirooka M., Koizumi Y., Miyake T., Ochi H., Tokumoto Y., Tada F., Matsuura B., Abe M., Hiasa Y. (2015). Nonalcoholic fatty liver disease: Portal hypertension due to outflow block in patients without cirrhosis. Radiology.

[46] Yeh M.M., Brunt E.M. (2014). Pathological features of fatty liver disease. Gastroenterology.

[47] Brunt E.M. (2016). Nonalcoholic fatty liver disease: Pros and cons of histologic systems of evaluation. Int. J. Mol. Sci..

[48] Sase S., Monden M., Oka H., Dono K., Fukuta T., Shibata I. (2002). Hepatic blood flow measurements with arterial and portal blood flow mapping in the human liver by means of xenon CT. J. Comput. Assist. Tomogr..

[49] Sase S., Takahashi H., Ikeda H., Kobayashi M., Matsumoto N., Suzuki M. (2008). Determination of time-course change rate for arterial xenon using the time course of tissue xenon concentration in xenonenhanced computed tomography. Med. Phys..

[50] Kuno A., Ikehara Y., Tanaka Y., Ito K., Matsuda A., Sekiya S., Hige S., Sakamoto M., Kage M., Mizokami M. (2013). A serum “sweet-doughnut” 272 protein facilitates fibrosis evaluation and therapy assessment in patients 273 with viral hepatitis. Sci. Rep..

